# Supplementary material for: Assessing the consequences of gestational diabetes mellitus on offspring’s cardiovascular health: MySweetHeart Cohort study protocol, Switzerland
Source: BMJ Open. 2017 Nov 14;7(11):e016972. doi: 10.1136/bmjopen-2017-016972 (PMC5695409; doi:10.1136/bmjopen-2017-016972)
Supplement: Author's manuscript [file bmjopen-2017-016972.draft_revisions.pdf]

BMJ Open is committed to open peer review. As part of this commitment we make the peer review history of every article we publish publicly available.

When an article is published we post the peer reviewers' comments and the authors' responses online. We also post the versions of the paper that were used during peer review. These are the versions that the peer review comments apply to.

The versions of the paper that follow are the versions that were submitted during the peer review process. They are not the versions of record or the final published versions. They should not be cited or distributed as the published version of this manuscript.

BMJ Open is an open access journal and the full, final, typeset and author-corrected version of record of the manuscript is available on our site with no access controls, subscription charges or pay-per-view fees (http://bmjopen.bmj.com).

If you have any questions on BMJ Open's open peer review process please email editorial.bmjopen@bmj.com

BMJ Open

Assessing the consequences of gestational diabetes mellitus on offspring's cardiovascular health: MySweetHeart Cohort study protocol.

Journal:	*BMJ Open*
Manuscript ID	bmjopen-2017-016972
Article Type:	Protocol
Date Submitted by the Author:	23-Mar-2017

Complete List of Authors:	Di Bernardo, Stefano; Lausanne University Hospital, Paediatric Cardiology Unit, Woman-Mother-Child Department<br>Mivelaz, Yvan; Lausanne University Hospital, Paediatric Cardiology Unit, Woman-Mother-Child Department<br>Epure, Adina; Lausanne University Hospital, Paediatric Cardiology Unit, Woman-Mother-Child Department ; Lausanne University Hospital, Institute of Social and Preventive Medicine<br>Vial, Yvan; Lausanne University Hospital, Obstetrics and Gynaecology Division, Woman-Mother-Child Department<br>Simeoni, Umberto; Lausanne University Hospital, DOHad Laboratory, Paediatrics Division, Woman-Mother-Child Department<br>Bovet, Pascal; Lausanne University Hospital, Institute of Social and Preventive Medicine<br>Estoppey, Sandrine; Lausanne University Hospital, Institute of Social and Preventive Medicine<br>Chiolero, Arnaud; Lausanne University Hospital, Institute of Social and Preventive Medicine; McGill University, Epidemiology<br>Sekarski, Nicole; Lausanne University Hospital, Paediatric Cardiology Unit, Woman-Mother-Child Department

**Primary Subject Heading**:	Cardiovascular medicine
Secondary Subject Heading:	Paediatrics, Diabetes and endocrinology, Epidemiology
Keywords:	Gestational Diabetes, Cardiovascular Disease, Carotid intima-media thickness, Left ventricular mass index, Foetal programming, Paediatric

SCHOLARONE™ Manuscripts

Assessing the consequences of gestational diabetes mellitus on offspring's cardiovascular health: *MySweetHeart Cohort* study protocol.

Stefano Di Bernardo[1*], Yvan Mivelaz[1*], Adina Mihaela Epure[1,2], Yvan Vial[3], Umberto Simeoni[4], Pascal Bovet[2], Sandrine Estoppey[2], Arnaud Chiolero[2,5,6], Nicole Sekarski[1] on behalf of *MySweetHeart Research Group* (collaborators listed at the end of the manuscript)

[1] Paediatric Cardiology Unit, Woman-Mother-Child Department, Lausanne University Hospital, Lausanne, Switzerland;
[2] Institute of Social and Preventive Medicine (IUMSP), Lausanne University Hospital, Lausanne, Switzerland;
[3] Obstetrics and Gynaecology Division, Woman-Mother-Child Department, Lausanne University Hospital, Lausanne, Switzerland;
[4] DOHad Laboratory, Paediatrics Division, Woman-Mother-Child Department, Lausanne University Hospital, Lausanne, Switzerland;
[5] Department of Epidemiology, Biostatistics, and Occupational Health, McGill University, Montreal Canada;
[6] Institute of Primary Health Care (BIHAM), University of Bern, Switzerland.

*These authors contributed equally to the present work.

Correspondence to:
Prof. Nicole Sekarski
Paediatric Cardiology Unit
Department Woman-Mother-Child
Lausanne University Hospital
BH 11 977
Rue du Bugnon 46
1011 Lausanne, Switzerland
email: nicole.sekarski@chuv.ch
Phone: +41 (0)21 314 35 55

Keywords: gestational diabetes, cardiovascular disease, carotid intima-media thickness, left ventricular mass index, foetal programming, newborn, paediatric

Word count (excluding title page, abstract, references, figures, tables and appendices): 3952
Number of tables: 1
Number of figures: 1
Number of references: 67
Number of supplementary files for online only publication: 0

For peer review only - http://bmjopen.bmj.com/site/about/guidelines.xhtml

ABSTRACT

Introduction

Gestational diabetes mellitus (GDM) is a state of glucose intolerance with onset during pregnancy. GDM carries pre- and perinatal risks as well as long-term risks for the mother and her child. GDM may be involved in the foetal programming of long-term cardiovascular health. However, evidence is sparse and the effect of GDM on cardiovascular health unknown. To address these issues, we will conduct *MySweetHeart Cohort study*. The objectives are to assess the effect of GDM on offspring's cardiovascular health early in life by using surrogate markers of cardiovascular disease and atherosclerosis.

Methods and analysis

This is a cohort study of 100 offspring of women with GDM and 100 offspring of women without GDM. At inclusion, a baseline assessment of the mothers will be conducted through means of self-report questionnaires, a researcher-administrated interview, blood pressure and anthropometric measurements, and a maternal blood sampling. Between the 30th and 34th weeks of gestation, a foetal echography will be performed to assess the foetal cardiac structure and function, the foeto-maternal circulation, and the hepatic volume. At birth, maternal and neonatal characteristics will be assessed. An echocardiography will be performed to assess cardiac structure and function 2-7 days after birth; carotid intima-media thickness will be also measured to assess vascular structure. *MySweetHeart Cohort* is linked to *MySweetHeart Trial* (clinicaltrials.gov/ct2/show/NCT02890693), a randomized controlled trial assessing the effect of a multidimensional interdisciplinary lifestyle and psychosocial intervention to improve the cardio-metabolic and mental health of women with GDM and their offspring. A long-term follow-up of children is planned.

Ethics and dissemination

Ethical approval has been obtained through the state Human Research Ethics Committee of the Canton de Vaud (study number 2016-00745). We aim to disseminate the findings through regional, national, and international conferences and through peer-reviewed journals.

Trial registration number: clinicaltrials.gov/ct2/show/NCT02872974.

For peer review only - http://bmjopen.bmj.com/site/about/guidelines.xhtml

STRENGTHS AND LIMITATIONS OF THIS STUDY

- This study will increase our understanding of the impact of gestational diabetes mellitus on cardio-metabolic health during foetal and neonatal periods. Within a Developmental Origin of Health and Disease (DOHaD) framework, it will contribute to improving the knowledge on the early life conditioning (programming) mechanisms and their influence on infants' cardiovascular health.
- A major strength of our study is the assessment at birth of surrogate markers of cardiovascular disease [left ventricular mass index and subclinical atherosclerosis (carotid intima media thickness)].
- Having a better understanding of the clinical impact of the maternal hyperglycaemic disorders on offspring's health early in life is needed to inform strategies for the early prevention of cardiovascular diseases.
- A long term follow-up of children is planned for further assessment of their cardiovascular health.

For peer review only

For peer review only - http://bmjopen.bmj.com/site/about/guidelines.xhtml

INTRODUCTION

Maternal hyperglycaemic disorders during pregnancy

The prevalence of maternal hyperglycaemic disorders during pregnancy is increasing in the population due to the obesity epidemic and the increasing age of pregnant mothers [1-3]. Maternal hyperglycaemic disorders include type 1 and 2 diabetes mellitus (T1/T2DM) and gestational diabetes mellitus (GDM). T1/T2DM is a state of glucose intolerance occurring independently of pregnancy while GDM is characterized by glucose intolerance that begins or is first diagnosed during pregnancy and usually resolves after delivery [4, 5]. GDM is associated with an increased risk of maternal metabolic syndrome and T2DM [2, 3, 6] and is now the most frequent form of hyperglycaemic disorders during pregnancy [2].

Maternal T1/T2DM diabetes is associated with significant foetal and neonatal morbidity [4], e.g., large for gestational age (LGA), congenital malformations, and neonatal metabolic disorder (hypoglycaemia) [3, 7, 8]. There is some evidence that the foetal consequences are more serious with T1/T2DM diabetes than with GDM [4] and, until recently, the consequences of GDM on offspring's health were uncertain [9]. Nevertheless, the large HAPO cohort study showed that maternal hyperglycaemia less severe than in T1/T2DM, after correction for potential confounders (maternal obesity, blood pressure), was also associated with adverse perinatal outcomes, i.e., LGA, neonatal hyperinsulinism and hypoglycaemia, and preeclampsia [9-11]. Furthermore, in large randomized trials, treatment of GDM resulted in better perinatal outcomes, including a reduced risk of LGA [4, 12-15].

Following the results of these studies, the International Association of Diabetes and Pregnancy Study Groups (IADPSG) have proposed new criteria to diagnose GDM [3]. The benefits of screening and treatment of GDM have long been debated [14-17] and recommendations from medical societies varied from no screening at all, screening only high-risk women (risk-based screening), to universal screening. Thus, the IADPSG recommend universal one-stage screening procedure that includes a 2-hour 75-g oral glucose tolerance test (OGTT). A single abnormal glucose concentration at fasting, at one hour or at two hours is sufficient for the diagnosis of GDM [14]. New blood glucose threshold were derived from the HAPO study and based on pregnancy outcomes associated with the glucose level [10, 14]. Using these criteria, the prevalence of GDM is expected to be high, reaching up to 10 to 20% of pregnant women in U.S. populations [3].

Cardio-metabolic effects of maternal DM

Maternal hyperglycaemic disorders are associated with foetal cardiac and vascular structural and functional alterations. For instance, it is well known that offspring of T1/T2DM mothers are at increased risk of congenital cardiac malformations and cardiac hypertrophy [8, 18]. In a retrospective study of 92 offspring of 87 diabetic mothers conducted in Lausanne, we observed that 5 neonates had congenital heart disease and 12 had ventricular hypertrophy [8]. There is also growing evidence that maternal hyperglycaemic disorders may be involved in the "foetal programming" of obesity and metabolic disorders in their offspring [11, 19]. Within a Developmental Origin of Health and Disease framework [20, 21], foetal programming (or conditioning) is the process involved in the associations between the exposure to detrimental factors during foetal life and health outcomes later in life [22]. Foetal programming is suspected to be involved in the development of cardio-metabolic disorders, such as elevated blood pressure, coronary heart diseases or DM, notably through epigenetic mechanisms [20, 21].

For peer review only - http://bmjopen.bmj.com/site/about/guidelines.xhtml

Despite recent and large growth in this research area, studies are needed to better objectively characterize both early life exposure and cardio-metabolic outcomes [23].

Several epidemiologic studies suggest that intrauterine environment of women with DM contribute to the long term risk of metabolic diseases and intergenerational transmission of cardio-metabolic risk [11, 24]. For example, adult offspring of women with DM during gestation have increased risk of overweight/obesity [24-27], metabolic syndrome [24, 26], T2DM [28, 29], or pre-diabetes [29]. Exposure to maternal impaired glucose tolerance or DM during foetal life is associated with large foetal fat mass [30] and overweight/obesity in childhood [31, 32]. A large sibling study showed that the association between maternal DM and offspring BMI was most likely due to in utero exposure to hyperglycaemia and not to confounding by shared familial characteristics [33]. It was also shown that offspring who had been exposed to maternal DM during foetal life exhibit higher prevalence of impaired glucose tolerance and markers of insulin resistance in childhood and adolescence [34, 35].

The association between maternal hyperglycaemic disorders and LGA seems to be due to the increase in foetal insulin production in response to the increased transplacental transfer of maternal glucose [4, 36]. In the HAPO study, maternal hyperglycaemia and foetal hyperinsulinism were associated with high birth weight [10]. Treatment of GDM can limit foetal overgrowth. Intrauterine exposure to high maternal blood glucose has also been associated with greater lean mass and adiposity among prepubertal offspring [37].

During foetal life, maternal diabetes is associated with foetal hyperinsulinism responsible of structural and functional changes affecting mostly the liver and the cardiovascular system. The liver is the major metabolic organ, considered as the metabolic brain and responsible for the distribution of the placental resources [38]. Recent studies have shown that macrosomic offspring had impaired liver blood perfusion that contributes to foetal growth alteration [39-41] and that differential perfusion of the foetal liver modified hepatic metabolic function and size [38]. Studies indicate that the liver is enlarged in foetuses of T1/T2 DM mothers [42, 43]. Furthermore, and similarly, the foetal heart response to hyperinsulinism is the development of an asymmetrical hypertrophy predominant at the septal wall. The evaluation of liver volume, interventricular septum thickness, and left ventricular mass (LVM) is therefore of outmost importance and may represent surrogate markers of the foetal metabolic response to maternal GDM.

Lack of data on the effects of GDM on offspring's cardiovascular health

Most studies on the effect of maternal hyperglycaemic disorders were conducted among mothers with T1/T2DM, not among mothers with GDM, and with an emphasis on the effects on offspring's metabolic outcomes rather than on offspring's cardiovascular health. It is also unknown whether treatment of GDM is effective to reduce long-term risk of offspring's metabolic and CVD risk [9, 11, 15] and the new definition of GDM calls also for the conduction of further studies on the impact of GDM on offspring's cardiovascular health. To study the early development of atherosclerosis and pathogenesis of CVD, it has become central to assess surrogate markers of CVD such as increased cardiac mass (left ventricular mass index; LVMI) [44] and of subclinical atherosclerosis (assessed through carotid intima-media thickness; cIMT) [44, 45]. These markers have been used extensively in studies in children and young adults with CVD risk factors [44-48]. However, to our knowledge, the effects of GDM on offspring's foetal and early neonatal cardiovascular health, and in particular on such surrogate markers [45], have never been studied.

For peer review only - http://bmjopen.bmj.com/site/about/guidelines.xhtml

Objectives

Our objectives are to assess the effect of GDM on offspring's cardiovascular health early in life, namely on (i) the surrogate markers of cardiovascular disease (CVD) and atherosclerosis at birth [left ventricular mass index (LVMI) and carotid intima-media thickness (cIMT)] (primary outcomes) and (ii) the cardiovascular structure and function during the foetal period and neonatal cardiovascular risk factors (secondary outcomes).

METHODS AND ANALYSIS

Study Design

We plan to conduct a cohort study of 200 offspring of women aged 18 years old or more. We will include 100 women with GDM and 100 without GDM. All pregnant women attending the antenatal care clinic or the GDM clinic at the Lausanne University Hospital (CHUV) will be invited to participate. In addition, women with GDM who are followed up by private diabetologists and gynaecologists in the Vaud Canton will be invited to participate following a similar procedure. Using the new criteria of the IADPSG, the expected prevalence of GDM in this population is at least ~10% [3]. Women with T1/T2D will not be included.

Study population

Inclusion criteria: Pregnant women aged 18 years or older, with or without GDM at 24-32 weeks of gestation, and understanding French or English; exclusion criteria: Women on strict bed-rest, with pre-existing DM or severe mental disorders precluding participation.

Study outcomes

Primary outcomes are surrogate markers of CVD and atherosclerosis at birth, that is, LVMI and cIMT. Secondary outcomes are foetal cardiovascular structure and function (notably LVMI and liver volume) and neonatal cardiovascular risk factors (including blood cord lipid level, glycaemia, insulin, hs-CRP, etc.).

Data collection and visits

During pregnancy, in the pre-recruitment phase, all pregnant women without pre-existing DM, attending the CHUV antenatal care clinic, have a fasting blood glucose measurement between the 24th and 28th weeks of gestation. GDM is diagnosed if fasting blood glucose is equal to or above 5.1 mmol/l. If fasting blood glucose is equal to or above 4.4 and below 5.1 mmol/l, pregnant women have a 2-hour 75-g OGTT and GDM will be diagnosed based on the new criteria of the IADPSG [3, 9]. Women screened by private obstetricians in the Vaud canton undergo either the same procedure or undergo directly a 2-hour 75-g OGTT. Considering the variability in patients' compliance and time of enrolment for prenatal care, all eligible women, having been screened for GDM before 32 weeks of gestation, will be invited to participate (see inclusion and exclusion criteria above).

The participation in the study is voluntary and involves a sequence of events and measurements as summarized in Table 1.

For peer review only - http://bmjopen.bmj.com/site/about/guidelines.xhtml

Table 1. Schedule of events and main assessments. WG: weeks of gestation. CVD: cardiovascular diseases, N/A: not applicable.

	Visit	Period (WG)	Mother	Foetus/Newborn
Pregnancy	V1 (Baseline)	24th-32nd	• Information notably on socio-economic characteristics, general health, family history of CVD or diabetes, and smoking and drinking habits<br>• Weight and height measurements<br>• 3 blood pressure measurements<br>• Blood sampling	• N/A
	V2	30th-34th	• Weight<br>• 3 blood pressure measurements	• Foetal cardiac echography
Delivery or Post-delivery	V3	Birth	• Blood sampling	• Cord blood sampling, newborn weight, length and other birth-related information
	V4	2-7 days after birth	• Weight<br>• 3 blood pressure measurements	• Neonatal cardiac echography and carotid echography

At the first study visit (V1), scheduled between the 24th and 32nd weeks of gestation, a baseline assessment of the mothers will be conducted through means of self-report questionnaires and a researcher-administrated interview, weight, height and blood pressure measurements, and a maternal blood sampling. This will provide information notably on the socioeconomic status, general health status, family medical history of CVD or diabetes, smoking and drinking habits, and several cardio-metabolic biomarkers, all of which will be treated as potential confounding factors. If available, paternal data on the family history of CVD or diabetes will be also collected at the first study visit.

At the second visit (V2), between the 30th and 34th weeks of gestation, a foetal echography will be performed to assess the foetal cardiac structure (including LVMI) and function, the foeto-maternal circulation, and the hepatic volume.

In women with GDM, information about treatment and blood glucose measurements will be recorded during the whole pregnancy.

At birth (V3), maternal characteristics (including mode of delivery, gravidity, parity, etc.) and neonatal characteristics (including gestational age, sex, weight, length, etc.) will be assessed. A sample of umbilical cord blood will be collected to measure various biomarkers including glucose, insulin, total cholesterol, LDL-cholesterol, HDL-cholesterol, triglycerides, uric acid, creatinine, hs-CRP, and various miRNA.

An echocardiography will be done to assess cardiac structure (including LVMI) and function 2-7 days after birth (V4). Carotid intima-media thickness (IMT) will be measured to assess vascular structure.

For peer review only - http://bmjopen.bmj.com/site/about/guidelines.xhtml

Measurements methods

Maternal clinical assessment

Maternal body weight and blood pressure [3 readings obtained at 2-min intervals with a clinically validated [49] oscillometric sphygmomanometer (OMRON HEM-907) [50, 51] will be measured during pregnancy (V1, V2) and in postpartum (V4). Clinical chemistry (including glucose, HbA1c, insulin, total cholesterol, LDL-cholesterol, HDL-cholesterol, triglycerides, uric acid, and creatinine) will be assessed at the first study visit (V1) and blood will be collected for further potential analyses. All samples will be managed and stored in a biobank. The clinical data assessed as part of the usual care of the mother and potentially influencing the outcomes of interest will be collected. Also, information on socio-economic characteristics, general health, family history of CVD or diabetes, smoking and drinking habits, etc., will be gathered using a self-report questionnaire and a researcher-administrated interview.

Offspring clinical assessment

Gestational age will be determined using either the date of the last menstrual period, or the first trimester ultrasound dating. Clinical chemistry (including glucose, total cholesterol, LDL-cholesterol, HDL-cholesterol, triglycerides, insulin, uric acid, hs-CRP, mi-RNA) will be assessed at birth using cord blood. Blood for future analyses will be stored in a biobank. The clinical data assessed as part of the usual care of the offspring and potentially influencing the outcomes of interest will be collected.

Foetal echography

This exam will be performed between the 30th and 34th weeks of gestation by two experienced ultrasonographers (YM, YV), blinded to the maternal glycaemic status, using a Voluson E10 (General Electric Medical Systems, ZIPF, Australia) equipped with a 2–8 MHz convex array sector transducer [52]. A systematic echography will be conducted to detect structural and functional anomalies following international recommendations [53, 54]. Anthropometric measurements and foetal well-being parameters will be measured. Then, a detailed echocardiography of the foetal liver, cardiovascular system and of the materno-foetal circulation will be conducted including bidimensional (2D), M-Mode (M), Doppler (D) and tridimensional (3D) imaging of the following structures: cardiac 4 chambers (2D), RV and LV short axis (2D, M), tricuspid, pulmonary, mitral and aortic valve (2D, D), umbilical vein, ductus venosus, patent ductus arteriosus, aortic isthmus, umbilical arteries and maternal uterine arteries (2D, D) and liver (2D, 3D). This will allow the assessment of the LV posterior wall and septal thickness, the LV mass [55], the LV and RV systolic and diastolic function, the cardiothoracic ratio, the placental and foetal vascular resistances, the cardiac output and liver volume. Data will be digitally stored to allow off-line analyses. Measures will be expressed as z-scores based on gestational age to allow comparison between foetuses.

Neonatal cardiac echography

This exam will be performed 2-7 days after birth (before the mother and the newborn leave the clinic) by two experienced ultrasonographers (NS, SDB) blinded to the maternal glycaemic status and to the foetal echocardiogram. Echocardiography will be performed on a Philips EPIC echocardiogram with a S8-3 or S12 MHz transducer, digitally recorded (Xcelera, Philips) and analyzed off-line. Standard echocardiography including 2D, M-mode, colour and spectral Doppler according to the ASE will be performed [56, 57]. Measurements will be performed

For peer review only - http://bmjopen.bmj.com/site/about/guidelines.xhtml

according to the standard of the ASE [56, 57] including: 1) right and left atrial chamber sizes; 2) LV size, volume and function; 3) LV mass; 4) LV and RV systolic and diastolic function. Z scores based on BSA calculated by the Haycock formula will be used to express these measurements [58-60].

Neonatal carotid echography

This exam will be performed 2-7 days after birth (before the mother and the newborn leave the clinic) by two experimented ultrasonographers (NS, SDB) blinded to the maternal glycaemic status. Carotid IMT measurement will be performed on a Philips EPIC echocardiograph (Philips Medical, Netherland) with a L 11-5 MHz high-resolution linear array transducer, recorded on a digital system (QLab, Philips Medical Netherlands). Image acquisition will be done according to the standard of the American Heart Association (AHA) Atherosclerosis, Hypertension and Obesity in Youth Committee of the Council on Cardiovascular Disease in the Young [45]. Image analyses will include: 1) far-wall cIMT; 2) calculation of the mean of maximal cIMT measurements; 3) calculation of carotid stiffness. Measurements of cIMT will be expressed in mm as mean +/- SD and compared with normal cIMTs [61, 62]. We have recently conducted a study to show that the measurement of cIMT was feasible in young non-sedated infants. This study conducted among 81 infants less than 1 year of age confirmed that cIMT was measurable with a high inter-observer reliability (coefficient of variation: 5.9%)[63].

Anticipated recruitment

Recruitment has begun in September 2016. We expect to include up to 2 pregnant women per week, hence to include the 200 subjects in 30 months. A research coordinator (SE) and a PhD student (AME) will be responsible to screen all women for eligibility and to ensure follow-up throughout the study duration. Recruitment will be facilitated by the high support of obstetricians at the CHUV. We will also benefit of the new specialized GDM clinic of the CHUV where every pregnant woman who screened positive for GDM at the CHUV antenatal care clinic is followed up. *MySweetHeart Cohort* is linked to *MySweetHeart Trial* (clinicaltrials.gov: NCT02890693; led by Prof Jardena Puder and PD Dr Antje Horsch), a randomized controlled trial led by this new specialized GDM clinic designed to assess the effect of a multidimensional interdisciplinary lifestyle and psychosocial intervention to improve the cardio-metabolic and mental health of women with GDM and their offspring.

Sample size

Assuming that LVMI will be 30 $g/m^{2.7}$ [SD: 4.5] in children of mothers without GDM [64] compared to 32 $g/m^{2.7}$ [SD: 4.5] in children of women with GDM, the required sample size is 80 women with GDM and 80 women without GDM to have a statistical significant difference with a power of 80% and an alpha-level set at 0.05 (two-sided). This sample size is also sufficient to observe statistical significant differences in cIMT at birth if we assume that cIMT at birth is 0.44 mm [SD: 0.04] [45] in newborn of women with GDM and 0.42 mm [SD: 0.04] in newborn of women without GDM. In the worst case scenario, we could lose up to 20% of participants between inclusion and delivery. Therefore, we plan to include 100 pregnant women with GDM and 100 women without GDM.

For peer review only - http://bmjopen.bmj.com/site/about/guidelines.xhtml

Statistical analysis

The association between exposure (GDM/no GDM) and the (continuous) outcomes will be estimated with linear regression analyses. Adjustment for potential confounding factors (maternal characteristics such as age, BMI, blood pressure, socio-economic status, personal history of CVD; treatment for GDM during pregnancy) will be done. To assess the direct and indirect (through mediators) effects of GDM on the outcomes, causal mediation analyses will be conducted [65, 66]. Because LGA is a potential mediator of the effect of GDM on offspring outcomes [67], analyses adjusted on birth weight will be conducted to estimate the indirect effect of GDM on the outcomes **(Figure 1)**. We will also consider the effect of treatment which will be treated as a mediator between GDM, LGA, and outcomes.

---FIGURE 1---

ETHICS AND DISSEMINATION

The study poses little to no risk to participants and their offspring. Signed informed consent is obtained from all participating women. Participation in the study does not interfere with the typical care patients receive during pregnancy and after delivery. Clinicians in the obstetrical clinic provide clinical follow-up and, if necessary, participants are referred on for additional clinical management.

Results from this study will be disseminated at regional and international conferences and in peer-reviewed journals.

SIGNIFICANCE AND OUTLOOK

The current high prevalence of maternal obesity and associated hyperglycaemic disorders may lead to a perpetuating intergenerational cycle of increasing obesity, metabolic and cardiovascular disorders. Well-designed prospective studies are necessary to better quantify the actual impact of maternal hyperglycaemic disorders on offspring's health and to identify the potential underlying mechanisms. Despite recent significant advances in Developmental Origin of Health and Disease and foetal programming research, major challenges have to be addressed, major challenges have to be addressed, notably by better objectively characterizing both early life exposure and cardio-metabolic outcomes [23].

Indeed, many past studies on the foetal programming of cardio-metabolic disorders suffered from major methodological flaws including confounding, inconsistent results, inadequate study design, no or poor standardization of exposure and outcome assessment, and selection bias. Our prospective population-based study in pregnant women whose offspring is examined during foetal life and at birth avoids these issues. The long period needed for CVD to develop could be a limitation, but this will be overcome in our study by measuring surrogate outcomes (including LVMI and cIMT) that are well-established precursors of CVD [45]. In addition, a long-term follow-up of the offspring is planned (not funded yet) for further assessment of their cardiovascular health.

By looking at the foeto-maternal circulation, foetal cardiovascular system and liver, and foetal growth and CVD risk markers at birth, we will acquire unique data to gain insight in the early mechanisms underlying the development of CVD and metabolic diseases, and on the role of

For peer review only - http://bmjopen.bmj.com/site/about/guidelines.xhtml

maternal hyperglycaemic disorders. GDM is an opportunity for timely intervention for the prevention of cardio-metabolic disorders in mothers and their offspring [9]. With the new criteria to define GDM, a growing proportion of women will be concerned by this disorder. Having a better understanding of the clinical impact of maternal hyperglycaemic disorders on offspring's health early in life is urgently needed to inform strategies for the early prevention of CVD.

Acknowledgements

Not applicable.

Collaborators

The following are members of *MySweetHeart Research Group* (including the authors of the present article), listed in alphabetical order: Pascal Bovet, MD MPH, Prof. (Institute of Social and Preventive Medicine (IUMSP), Lausanne University Hospital, Lausanne, Switzerland; pascal.bovet@chuv.ch), Arnaud Chiolero, MD PhD, Senior lecturer (Institute of Social and Preventive Medicine (IUMSP), Lausanne University Hospital, Lausanne, Switzerland; Department of Epidemiology, Biostatistics, and Occupational Health, McGill University, Montreal Canada; Institute of Primary Health Care (BIHAM), University of Bern, Switzerland; arnaud.chiolero@chuv.ch), Stefano Di Bernardo, MD, Lecturer (Pediatric Cardiology Unit, Woman-Mother-Child Department, Lausanne University Hospital, Lausanne, Switzerland; stefano.di-bernardo@chuv.ch), Adina Mihaela Epure, MD (Pediatric Cardiology Unit, Woman-Mother-Child Department; Institute of Social and Preventive Medicine (IUMSP), Lausanne University Hospital, Lausanne, Switzerland; adina-mihaela.epure@chuv.ch), Sandrine Estoppey, M.Sc. (Institute of Social and Preventive Medicine (IUMSP), Lausanne University Hospital, Lausanne, Switzerland; sandrine.estoppey@chuv.ch), Leah Gilbert, M.Sc. (Division of Endocrinology, Diabetes and Metabolism, Department of Medicine, Lausanne University Hospital, Lausanne, Switzerland; leah.gilbert@chuv.ch), Justine Gross, HES (Division of Endocrinology, Diabetes and Metabolism, Department of Medicine, Lausanne University Hospital, Lausanne, Switzerland; justine.gross@chuv.ch), Antje Horsch, DClinPsych, Senior Lecturer, Woman-Mother-Child Department, Lausanne University Hospital, Lausanne, Switzerland; antje.horsch@chuv.ch), Bengt Kayser, PhD, Prof. (Institute of Sport Sciences, University of Lausanne; bengt.kayser@unil.ch), Stefano Lanzi, PhD (Division of Endocrinology, Diabetes and Metabolism, Department of Medicine, Lausanne University Hospital, Lausanne, Switzerland; stefano.lanzi@chuv.ch), Yvan Mivelaz, MD, Lecturer (Pediatric Cardiology Unit, Woman-Mother-Child Department, Lausanne University Hospital, Lausanne, Switzerland; yvan.mivelaz@chuv.ch), Jardena J. Puder, MD, Prof. (Division of Endocrinology, Diabetes and Metabolism, Department of Medicine, Lausanne University Hospital, Lausanne, Switzerland; jardena.puder@chuv.ch), Nicole Sekarski, MD, Prof. (Pediatric Cardiology Unit, Woman-Mother-Child Department, Lausanne University Hospital, Lausanne, Switzerland; nicole.sekarski@chuv.ch), Umberto Simeoni, MD, Prof. (DOHad Laboratory, Pediatrics Division, Woman-Mother-Child Department, Lausanne University Hospital, Lausanne, Switzerland; umberto.simeoni@chuv.ch), Yvan Vial, MD, Senior lecturer (Obstetrics and Gynecology Division, Woman-Mother-Child Department, Lausanne University Hospital, Lausanne, Switzerland; yvan.vial@chuv.ch).

Contributors

NS, AC, SDB and YM designed the study with input from all other authors. SDB and YM drafted the manuscript and contributed equally to the present work. US, PB, AME, SE, YV, and AC

For peer review only - http://bmjopen.bmj.com/site/about/guidelines.xhtml

significantly contributed to the establishment and refinement of study procedures and critically revised the manuscript. All authors approved the final version of the manuscript.

Funding

This study is funded by the Swiss National Science Foundation (Project 32003B-163240). The study protocol has been peer-reviewed by national and international experts before being funded.

Competing interests

None declared.

Ethics approval

The study has been approved by the state Human Research Ethics Committee of the Canton de Vaud (study number 2016-00745).

Provenance and peer review

Not commissioned; externally peer reviewed.

Data sharing statement

It is the intention of the authors that once *MySweetHeart Cohort* is completed and the research articles emerging from the study have been published, the data set supporting the results and conclusions presented in the respective research articles to be made available in the DATA@IUMSP repository, https://data.iumsp.ch.

Open access

This is an Open Access article distributed in accordance with the terms of the Creative Commons Attribution (CC BY 4.0) license, which permits others to distribute, remix, adapt and build upon this work, for commercial use, provided the original work is properly cited. See: http://creativecommons.org/licenses/by/4.0/

For peer review only

FIGURE LEGEND

Figure 1. Hypothetical and highly-simplified causal relationships between gestational diabetes mellitus (GDM) and cardio-metabolic disorders. Two pathways can be hypothesized: 1) a direct effect of GDM on offspring's cardio-metabolic disorders; 2) an indirect effect through the mediator large for gestational age (LGA). It means that if LGA was prevented, part of the effect of GDM on cardio-metabolic disorders would be prevented. Several confounding factors (such as maternal obesity, weight gain during pregnancy, smoking, socio-economic status, or family history of cardio-metabolic diseases) have to be accounted.

For peer review only

For peer review only - http://bmjopen.bmj.com/site/about/guidelines.xhtml

REFERENCES

1 Hunt KJ, Schuller KL. The increasing prevalence of diabetes in pregnancy. *Obstet Gynecol Clin North Am* 2007;34(2):173-99.
2 Albrecht SS, Kuklina EV, Bansil P, et al. Diabetes trends among delivery hospitalizations in the U.S., 1994-2004. *Diabetes Care* 2010;33(4):768-73.
3 Metzger BE, Gabbe SG, Persson B, et al. International association of diabetes and pregnancy study groups recommendations on the diagnosis and classification of hyperglycemia in pregnancy. *Diabetes Care* 2010;33(3):676-82.
4 Reece EA, Leguizamon G, Wiznitzer A. Gestational diabetes: the need for a common ground. *Lancet* 2009;373(9677):1789-97.
5 Reece EA. The fetal and maternal consequences of gestational diabetes mellitus. *J Matern Fetal Neonatal Med* 2010;23(3):199-203.
6 Noussitou P, Monbaron D, Vial Y, et al. Gestational diabetes mellitus and the risk of metabolic syndrome: a population-based study in Lausanne, Switzerland. *Diabetes Metab* 2005;31(4 Pt 1):361-9.
7 Landon MB, Mele L, Spong CY, et al. The relationship between maternal glycemia and perinatal outcome. *Obstet Gynecol* 2011;117(2 Pt 1):218-24.
8 Ullmo S, Vial Y, Di Bernardo S, et al. Pathologic ventricular hypertrophy in the offspring of diabetic mothers: a retrospective study. *Eur Heart J* 2007;28(11):1319-25.
9 Nolan CJ. Controversies in gestational diabetes. *Best Pract Res Clin Obstet Gynaecol* 2011;25(1):37-49.
10 Metzger BE, Lowe LP, Dyer AR, et al. Hyperglycemia and adverse pregnancy outcomes. *N Engl J Med* 2008;358(19):1991-2002.
11 Nolan CJ, Damm P, Prentki M. Type 2 diabetes across generations: from pathophysiology to prevention and management. *Lancet* 2011;378(9786):169-81.
12 Crowther CA, Hiller JE, Moss JR, et al. Effect of treatment of gestational diabetes mellitus on pregnancy outcomes. *N Engl J Med* 2005;352(24):2477-86.
13 Landon MB, Spong CY, Thom E, et al. A multicenter, randomized trial of treatment for mild gestational diabetes. *N Engl J Med* 2009;361(14):1339-48.
14 Leary J, Pettitt DJ, Jovanovic L. Gestational diabetes guidelines in a HAPO world. *Best Pract Res Clin Endocrinol Metab* 2010;24(4):673-85.
15 Landon MB. Is there a benefit to the treatment of mild gestational diabetes mellitus? *Am J Obstet Gynecol* 2010;202(6):649-53.
16 Hillier TA, Vesco KK, Pedula KL, et al. Screening for gestational diabetes mellitus: a systematic review for the U.S. Preventive Services Task Force. *Ann Intern Med* 2008;148(10):766-75.
17 Tieu J, Middleton P, McPhee AJ, et al. Screening and subsequent management for gestational diabetes for improving maternal and infant health. *Cochrane Database Syst Rev* 2010(7):CD007222.
18 Veille JC, Hanson R, Sivakoff M, et al. Fetal cardiac size in normal, intrauterine growth retarded, and diabetic pregnancies. *Am J Perinatol* 1993;10(4):275-9.
19 Yajnik CS. Fetal programming of diabetes: still so much to learn! *Diabetes Care* 2010;33(5):1146-8.
20 Hanson MA, Gluckman PD. Early developmental conditioning of later health and disease: physiology or pathophysiology? *Physiol Rev* 2014;94(4):1027-76.
21 Simeoni U, Yzydorczyk C, Siddeek B, et al. Epigenetics and neonatal nutrition. *Early Hum Dev* 2014;90 Suppl 2:S23-4.
22 Gluckman PD, Hanson MA, Cooper C, et al. Effect of in utero and early-life conditions on adult health and disease. *N Engl J Med* 2008;359(1):61-73.

For peer review only - http://bmjopen.bmj.com/site/about/guidelines.xhtml

For peer review only

23 Hivert MF, Perng W, Watkins SM, et al. Metabolomics in the developmental origins of obesity and its cardiometabolic consequences. *J Dev Orig Health Dis* 2015:1-14.
24 Clausen TD, Mathiesen ER, Hansen T, et al. Overweight and the metabolic syndrome in adult offspring of women with diet-treated gestational diabetes mellitus or type 1 diabetes. *J Clin Endocrinol Metab* 2009;94(7):2464-70.
25 Pettitt DJ, Baird HR, Aleck KA, et al. Excessive obesity in offspring of Pima Indian women with diabetes during pregnancy. *N Engl J Med* 1983;308(5):242-5.
26 Moore TR. Fetal exposure to gestational diabetes contributes to subsequent adult metabolic syndrome. *Am J Obstet Gynecol* 2010;202(6):643-9.
27 Lawlor DA, Lichtenstein P, Langstrom N. Association of maternal diabetes mellitus in pregnancy with offspring adiposity into early adulthood: sibling study in a prospective cohort of 280,866 men from 248,293 families. *Circulation* 2011;123(3):258-65.
28 Franks PW, Looker HC, Kobes S, et al. Gestational glucose tolerance and risk of type 2 diabetes in young Pima Indian offspring. *Diabetes* 2006;55(2):460-5.
29 Clausen TD, Mathiesen ER, Hansen T, et al. High prevalence of type 2 diabetes and pre-diabetes in adult offspring of women with gestational diabetes mellitus or type 1 diabetes: the role of intrauterine hyperglycemia. *Diabetes Care* 2008;31(2):340-6.
30 Parretti E, Carignani L, Cioni R, et al. Sonographic evaluation of fetal growth and body composition in women with different degrees of normal glucose metabolism. *Diabetes Care* 2003;26(10):2741-8.
31 Crume TL, Ogden L, Daniels S, et al. The impact of in utero exposure to diabetes on childhood body mass index growth trajectories: the EPOCH study. *J Pediatr* 2011;158(6):941-6.
32 Lawlor DA, Fraser A, Lindsay RS, et al. Association of existing diabetes, gestational diabetes and glycosuria in pregnancy with macrosomia and offspring body mass index, waist and fat mass in later childhood: findings from a prospective pregnancy cohort. *Diabetologia* 2010;53(1):89-97.
33 Lawlor DA, Lichtenstein P, Fraser A, et al. Does maternal weight gain in pregnancy have long-term effects on offspring adiposity? A sibling study in a prospective cohort of 146,894 men from 136,050 families. *Am J Clin Nutr* 2011;94(1):142-8.
34 Fetita LS, Sobngwi E, Serradas P, et al. Consequences of fetal exposure to maternal diabetes in offspring. *J Clin Endocrinol Metab* 2006;91(10):3718-24.
35 Burguet A. Long-term outcome in children of mothers with gestational diabetes. *Diabetes Metab* 2010;36(6 Pt 2):682-94.
36 Mitanchez D, Burguet A, Simeoni U. Infants born to mothers with gestational diabetes mellitus: mild neonatal effects, a long-term threat to global health. *J Pediatr* 2014;164(3):445-50.
37 Chandler-Laney PC, Bush NC, Rouse DJ, et al. Maternal glucose concentration during pregnancy predicts fat and lean mass of prepubertal offspring. *Diabetes Care* 2011;34(3):741-5.
38 Kiserud T. Diabetes in pregnancy: scanning the wrong horizon? *Ultrasound Obstet Gynecol* 2010;36(3):266-7.
39 Kessler J, Rasmussen S, Godfrey K, et al. Venous liver blood flow and regulation of human fetal growth: evidence from macrosomic fetuses. *Am J Obstet Gynecol* 2011;204(5):429 e1-7.
40 Ebbing C, Rasmussen S, Kiserud T. Fetal hemodynamic development of macrosomic growth. *Ultrasound Obstet Gynecol* 2011;38(3):303-8.
41 Tchirikov M, Kertschanska S, Sturenberg HJ, et al. Liver blood perfusion as a possible instrument for fetal growth regulation. *Placenta* 2002;23 Suppl A:S153-8.
42 Mirghani H, Zayed R, Thomas L, et al. Gestational diabetes mellitus: fetal liver length measurements between 21and 24 weeks' gestation. *J Clin Ultrasound* 2007;35(1):34-7.
43 Anderson NG, Notley E, Graham P, et al. Reproducibility of sonographic assessment of fetal liver length in diabetic pregnancies. *Ultrasound Obstet Gynecol* 2008;31(5):529-34.
44 Sorof JM, Alexandrov AV, Cardwell G, et al. Carotid artery intimal-medial thickness and left ventricular hypertrophy in children with elevated blood pressure. *Pediatrics* 2003;111(1):61-6.

For peer review only - http://bmjopen.bmj.com/site/about/guidelines.xhtml

45 Urbina EM, Williams RV, Alpert BS, et al. Noninvasive assessment of subclinical atherosclerosis in children and adolescents: recommendations for standard assessment for clinical research: a scientific statement from the American Heart Association. *Hypertension* 2009;54(5):919-50.
46 McGill HC, Jr., McMahan CA, Gidding SS. Preventing heart disease in the 21st century: implications of the Pathobiological Determinants of Atherosclerosis in Youth (PDAY) study. *Circulation* 2008;117(9):1216-27.
47 McGill HC, McMahan CA, Gidding SS. Are pediatricians responsible for prevention of adult cardiovascular disease? *Nat Clin Pract Cardiovasc Med* 2009;6(1):10-1.
48 Simonetti GD, RO VONV, Wuhl E, et al. Ambulatory arterial stiffness index is increased in hypertensive childhood disease. *Pediatr Res* 2008;64(3):303-7.
49 Chiolero A, Paradis G, Lambert M. Accuracy of oscillometric devices in children and adults. *Blood Press* 2010;19(4):254-9.
50 El Assaad MA, Topouchian JA, Darne BM, et al. Validation of the Omron HEM-907 device for blood pressure measurement. *Blood Press Monit* 2002;7(4):237-41.
51 White WB, Anwar YA. Evaluation of the overall efficacy of the Omron office digital blood pressure HEM-907 monitor in adults. *Blood Press Monit* 2001;6(2):107-10.
52 Salomon LJ, Alfirevic Z, Berghella V, et al. Practice guidelines for performance of the routine mid-trimester fetal ultrasound scan. *Ultrasound Obstet Gynecol* 2011;37(1):116-26.
53 Lee W, Allan L, Carvalho JS, et al. ISUOG consensus statement: what constitutes a fetal echocardiogram? *Ultrasound Obstet Gynecol* 2008;32(2):239-42.
54 Tutschek B, Schmidt KG. Techniques for assessing cardiac output and fetal cardiac function. *Semin Fetal Neonatal Med* 2011;16(1):13-21.
55 Reichek N, Helak J, Plappert T, et al. Anatomic validation of left ventricular mass estimates from clinical two-dimensional echocardiography: initial results. *Circulation* 1983;67(2):348-52.
56 Lai WW, Geva T, Shirali GS, et al. Guidelines and standards for performance of a pediatric echocardiogram: a report from the Task Force of the Pediatric Council of the American Society of Echocardiography. *J Am Soc Echocardiogr* 2006;19(12):1413-30.
57 Lopez L, Colan SD, Frommelt PC, et al. Recommendations for quantification methods during the performance of a pediatric echocardiogram: a report from the Pediatric Measurements Writing Group of the American Society of Echocardiography Pediatric and Congenital Heart Disease Council. *J Am Soc Echocardiogr* 2010;23(5):465-95; quiz 576-7.
58 Daubeney PE, Blackstone EH, Weintraub RG, et al. Relationship of the dimension of cardiac structures to body size: an echocardiographic study in normal infants and children. *Cardiol Young* 1999;9(4):402-10.
59 Pettersen MD, Du W, Skeens ME, et al. Regression equations for calculation of z scores of cardiac structures in a large cohort of healthy infants, children, and adolescents: an echocardiographic study. *J Am Soc Echocardiogr* 2008;21(8):922-34.
60 Haycock GB, Schwartz GJ, Wisotsky DH. Geometric method for measuring body surface area: a height-weight formula validated in infants, children, and adults. *J Pediatr* 1978;93(1):62-6.
61 Sass C, Herbeth B, Chapet O, et al. Intima-media thickness and diameter of carotid and femoral arteries in children, adolescents and adults from the Stanislas cohort: effect of age, sex, anthropometry and blood pressure. *J Hypertens* 1998;16(11):1593-602.
62 Jourdan C, Wuhl E, Litwin M, et al. Normative values for intima-media thickness and distensibility of large arteries in healthy adolescents. *J Hypertens* 2005;23(9):1707-15.
63 Mivelaz Y, Di Bernardo S, Boulos Ksontini T, et al. Feasibility and reliability of carotid intima-media thickness measurements in nonsedated infants. *J Hypertens* 2016;34(11):2227-32.
64 Maggio AB, Aggoun Y, Marchand LM, et al. Associations among obesity, blood pressure, and left ventricular mass. *J Pediatr* 2008;152(4):489-93.

For peer review only - http://bmjopen.bmj.com/site/about/guidelines.xhtml

65 VanderWeele TJ, Mumford SL, Schisterman EF. Conditioning on intermediates in perinatal epidemiology. *Epidemiology* 2012;23(1):1-9.
66 Chiolero A, Kaufman JS, Paradis G. Why adjustment for current weight can bias the estimate of the effect of birth weight on blood pressure: shedding light using causal graphs. *J Hypertens* 2012;30(5):1042-5.
67 Vohr BR, Boney CM. Gestational diabetes: the forerunner for the development of maternal and childhood obesity and metabolic syndrome? *J Matern Fetal Neonatal Med* 2008;21(3):149-57.

For peer review only

For peer review only - http://bmjopen.bmj.com/site/about/guidelines.xhtml

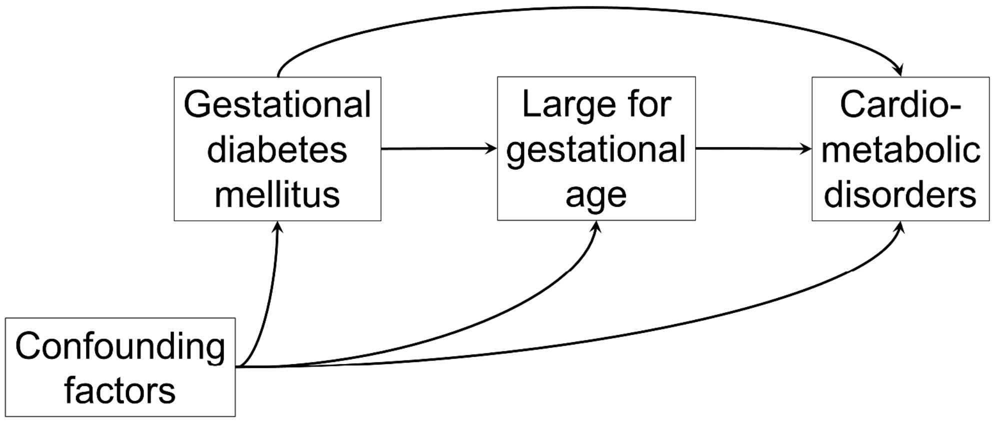

Hypothetical and highly-simplified causal relationships between gestational diabetes mellitus (GDM) and cardio-metabolic disorders. Two pathways can be hypothesized: 1) a direct effect of GDM on offspring's cardio-metabolic disorders; 2) an indirect effect through the mediator large for gestational age (LGA). It means that if LGA was prevented, part of the effect of GDM on cardio-metabolic disorders would be prevented. Several confounding factors (such as maternal obesity, weight gain during pregnancy, smoking, socio-economic status, or family history of cardio-metabolic diseases) have to be accounted.

338x190mm (300 x 300 DPI)

For peer review only - http://bmjopen.bmj.com/site/about/guidelines.xhtml

Assessing the consequences of gestational diabetes mellitus on offspring's cardiovascular health: MySweetHeart Cohort study protocol, Switzerland.

Journal:	*BMJ Open*
Manuscript ID	bmjopen-2017-016972.R1
Article Type:	Protocol
Date Submitted by the Author:	24-Jul-2017

Complete List of Authors:	Di Bernardo, Stefano; Lausanne University Hospital, Paediatric Cardiology Unit, Woman-Mother-Child Department<br>Mivelaz, Yvan; Lausanne University Hospital, Paediatric Cardiology Unit, Woman-Mother-Child Department<br>Epure, Adina Mihaela; Lausanne University Hospital, Paediatric Cardiology Unit, Woman-Mother-Child Department ; Lausanne University Hospital, Institute of Social and Preventive Medicine<br>Vial, Yvan; Lausanne University Hospital, Obstetrics and Gynaecology Division, Woman-Mother-Child Department<br>Simeoni, Umberto; Lausanne University Hospital, DOHad Laboratory, Paediatrics Division, Woman-Mother-Child Department<br>Bovet, Pascal; Lausanne University Hospital, Institute of Social and Preventive Medicine<br>Estoppey, Sandrine; Lausanne University Hospital, Institute of Social and Preventive Medicine<br>Chiolero, Arnaud; Lausanne University Hospital, Institute of Social and Preventive Medicine; McGill University, Epidemiology<br>Sekarski, Nicole; Lausanne University Hospital, Paediatric Cardiology Unit, Woman-Mother-Child Department

**Primary Subject Heading**:	Cardiovascular medicine
Secondary Subject Heading:	Paediatrics, Diabetes and endocrinology, Epidemiology
Keywords:	Gestational Diabetes, Cardiovascular Disease, Carotid intima-media thickness, Left ventricular mass index, Foetal programming, Paediatric

SCHOLARONE™ Manuscripts

Assessing the consequences of gestational diabetes mellitus on offspring's cardiovascular health: *MySweetHeart Cohort* study protocol, Switzerland.

Stefano Di Bernardo[1*], Yvan Mivelaz[1*], Adina Mihaela Epure[1,2], Yvan Vial[3], Umberto Simeoni[4], Pascal Bovet[2], Sandrine Estoppey[2], Arnaud Chiolero[2,5,6], Nicole Sekarski[1] on behalf of *MySweetHeart Research Group* (collaborators listed at the end of the manuscript)

[1] Paediatric Cardiology Unit, Woman-Mother-Child Department, Lausanne University Hospital, Lausanne, Switzerland;
[2] Institute of Social and Preventive Medicine (IUMSP), Lausanne University Hospital, Lausanne, Switzerland;
[3] Obstetrics and Gynaecology Division, Woman-Mother-Child Department, Lausanne University Hospital, Lausanne, Switzerland;
[4] DOHad Laboratory, Paediatrics Division, Woman-Mother-Child Department, Lausanne University Hospital, Lausanne, Switzerland;
[5] Department of Epidemiology, Biostatistics, and Occupational Health, McGill University, Montreal Canada;
[6] Institute of Primary Health Care (BIHAM), University of Bern, Switzerland.

*These authors contributed equally to the present work.

Correspondence to:
Prof. Nicole Sekarski
Paediatric Cardiology Unit
Woman-Mother-Child Department
Lausanne University Hospital
BH 11 977
Rue du Bugnon 46
1011 Lausanne, Switzerland
email: nicole.sekarski@chuv.ch
Phone: +41 (0)21 314 35 55

Keywords: gestational diabetes, cardiovascular disease, carotid intima-media thickness, left ventricular mass index, foetal programming, newborn, paediatric

Word count (excluding title page, abstract, references, figures, tables and appendices): 3935
Number of tables: 1
Number of figures: 1
Number of references: 63
Number of supplementary files for online only publication: 0

For peer review only

ABSTRACT

Introduction

Gestational diabetes mellitus (GDM) is a state of glucose intolerance with onset during pregnancy. GDM carries pre- and perinatal risks as well as long-term risks for the mother and her child. GDM may be involved in the foetal programming of long-term cardiovascular health. However, evidence is sparse and the effect of GDM on cardiovascular health unknown. To address these issues, we will conduct *MySweetHeart Cohort study*. The objectives are to assess the effect of GDM on offspring's cardiovascular health early in life by using surrogate markers of cardiovascular disease and atherosclerosis.

Methods and analysis

This is a cohort study of 100 offspring of women with GDM and 100 offspring of women without GDM. At inclusion, a baseline assessment of the mothers will be conducted through means of self-report questionnaires, a researcher-administrated interview, blood pressure and anthropometric measurements, and a maternal blood sampling. Between the 30th and 34th weeks of gestation, a foetal echography will be performed to assess the foetal cardiac structure and function, the foeto-maternal circulation, and the hepatic volume. At birth, maternal and neonatal characteristics will be assessed. An echocardiography will be performed to assess cardiac structure and function 2-7 days after birth; carotid intima-media thickness will be also measured to assess vascular structure. *MySweetHeart Cohort* is linked to *MySweetHeart Trial* (clinicaltrials.gov/ct2/show/NCT02890693), a randomized controlled trial assessing the effect of a multidimensional interdisciplinary lifestyle and psychosocial intervention to improve the cardio-metabolic and mental health of women with GDM and their offspring. A long-term follow-up of children is planned.

Ethics and dissemination

Ethical approval has been obtained through the state Human Research Ethics Committee of the Canton de Vaud (study number 2016-00745). We aim to disseminate the findings through regional, national, and international conferences and through peer-reviewed journals.

Trial registration number: clinicaltrials.gov/ct2/show/NCT02872974.

For peer review only - http://bmjopen.bmj.com/site/about/guidelines.xhtml

STRENGTHS AND LIMITATIONS OF THIS STUDY

- This study will increase our understanding of the impact of gestational diabetes mellitus on cardio-metabolic health during foetal and neonatal periods. Within a Developmental Origin of Health and Disease (DOHaD) framework, it will contribute to improving the knowledge on the early life conditioning (programming) mechanisms and their influence on infants' cardiovascular health.
- A major strength of our study is the assessment at birth of surrogate markers of cardiovascular disease [left ventricular mass index and subclinical atherosclerosis (carotid intima media thickness)].
- Acknowledged limitations are the relatively small sample size, the potential for residual confounding, and the limited representativeness of our sample (monocentric study).
- A better understanding of the clinical impact of the maternal hyperglycaemic disorders on offspring's health early in life is needed to inform strategies for the early prevention of cardiovascular diseases.
- A long term follow-up of children is planned for further assessment of their cardiovascular health.

For peer review only

For peer review only - http://bmjopen.bmj.com/site/about/guidelines.xhtml

INTRODUCTION

Maternal hyperglycaemic disorders during pregnancy

The prevalence of maternal hyperglycaemic disorders during pregnancy is increasing in the population due to the obesity epidemic and the increasing age of pregnant mothers [1-3]. Maternal hyperglycaemic disorders include type 1 and 2 diabetes mellitus (T1/T2DM) and gestational diabetes mellitus (GDM). T1/T2DM is a state of glucose intolerance occurring independently of pregnancy while GDM is characterized by glucose intolerance that begins or is first diagnosed during pregnancy and usually resolves after delivery [4, 5]. GDM is associated with an increased risk of maternal metabolic syndrome and T2DM [2, 3, 6] and is now the most frequent form of hyperglycaemic disorders during pregnancy [2].

Maternal T1/T2DM diabetes is associated with significant foetal and neonatal morbidity [4], e.g., large for gestational age (LGA), congenital malformations, and neonatal metabolic disorder (hypoglycaemia) [3, 7, 8]. There is some evidence that the foetal consequences are more serious with T1/T2DM diabetes than with GDM [4] and, until recently, the consequences of GDM on offspring's health were uncertain [9]. Nevertheless, the large HAPO cohort study showed that maternal hyperglycaemia less severe than in T1/T2DM, after correction for potential confounders (maternal obesity, blood pressure), was also associated with adverse perinatal outcomes, i.e., LGA, neonatal hyperinsulinism and hypoglycaemia, and preeclampsia [9-11]. Furthermore, in large randomized trials, treatment of GDM resulted in better perinatal outcomes, including a reduced risk of LGA [4, 12-15].

Following the results of these studies, the International Association of Diabetes and Pregnancy Study Groups (IADPSG) have proposed new criteria to diagnose GDM [3]. The benefits of screening and treatment of GDM have long been debated [14-17] and recommendations from medical societies varied from no screening at all, screening only high-risk women (risk-based screening), to universal screening. Thus, the IADPSG recommend universal one-stage screening procedure that includes a 2-hour 75-g oral glucose tolerance test (OGTT). A single abnormal glucose concentration at fasting, at one hour or at two hours is sufficient for the diagnosis of GDM [14]. New blood glucose threshold were derived from the HAPO study and based on pregnancy outcomes associated with the glucose level [10, 14]. Using these criteria, the prevalence of GDM is expected to be high, reaching up to 10 to 20% of pregnant women in U.S. populations [3].

Cardio-metabolic effects of maternal DM

Maternal hyperglycaemic disorders are associated with foetal cardiac and vascular structural and functional alterations. For instance, it is well known that offspring of T1/T2DM mothers are at increased risk of congenital cardiac malformations and cardiac hypertrophy [8, 18]. In a retrospective study of 92 offspring of 87 diabetic mothers conducted in Lausanne, we observed that 5 neonates had congenital heart disease and 12 had ventricular hypertrophy [8]. There is also growing evidence that maternal hyperglycaemic disorders may be involved in the "foetal programming" of obesity and metabolic disorders in their offspring [11, 19]. Within a Developmental Origin of Health and Disease framework [20, 21], foetal programming (or conditioning) is the process involved in the associations between the exposure to detrimental factors during foetal life and health outcomes later in life [22]. Foetal programming is suspected to be involved in the development of cardio-metabolic disorders, such as elevated blood pressure, coronary heart diseases or DM, notably through epigenetic mechanisms [20, 21].

For peer review only - http://bmjopen.bmj.com/site/about/guidelines.xhtml

Despite recent and large growth in this research area, studies are needed to better objectively characterize both early life exposures and cardio-metabolic outcomes [23].

Intrauterine exposure to high maternal blood glucose is associated with foetal hyperinsulinism which is responsible for structural and functional changes affecting mostly the liver and the cardiovascular system. The liver is the major metabolic organ, considered as the metabolic brain and responsible for the distribution of the placental resources [24]. Recent studies have shown that macrosomic offspring had impaired liver blood perfusion that contributes to foetal growth alteration [25-27] and that differential perfusion of the foetal liver modified hepatic metabolic function and size [24]. Studies indicate that the liver is enlarged in foetuses of T1/T2 DM mothers [28, 29]. Furthermore, and similarly, the foetal heart response to hyperinsulinism is the development of an asymmetrical hypertrophy predominant at the septal wall. The evaluation of liver volume, interventricular septum thickness, and left ventricular mass (LVM) is therefore of outmost importance and may represent surrogate markers of the foetal metabolic response to maternal GDM.

Lack of data on the effects of GDM on offspring's cardiovascular health

Most studies on the effect of maternal hyperglycaemic disorders were conducted among mothers with T1/T2DM, not among mothers with GDM, and with an emphasis on the effects on offspring's metabolic outcomes rather than on offspring's cardiovascular health. It is also unknown whether treatment of GDM is effective to reduce long-term risk of offspring's metabolic and CVD risk [9, 11, 15] and the new definition of GDM calls also for the conduction of further studies on the impact of GDM on offspring's cardiovascular health. To study the early development of atherosclerosis and pathogenesis of CVD, it has become central to assess surrogate markers of CVD such as increased cardiac mass (left ventricular mass index; LVMI) [30] and of subclinical atherosclerosis (assessed through carotid intima-media thickness; cIMT) [30, 31]. These markers have been used extensively in studies in children and young adults with CVD risk factors [30-34]. However, to our knowledge, the effects of GDM on offspring's foetal and early neonatal cardiovascular health, and in particular on such surrogate markers [31], have never been studied.

Objectives

Our objectives are to assess the effect of GDM on offspring's cardiovascular health early in life, namely on (i) the surrogate markers of cardiovascular disease (CVD) and atherosclerosis at birth [left ventricular mass index (LVMI) and carotid intima-media thickness (cIMT)] (primary outcomes) and (ii) the cardiovascular structure and function during the foetal period and potential neonatal cardiovascular risk factors (secondary outcomes).

METHODS AND ANALYSIS

Study Design

We plan to conduct a cohort study of 200 offspring of women aged 18 years old or more. We will include 100 women with GDM and 100 without GDM. All pregnant women attending the antenatal care clinic or the GDM clinic at the Lausanne University Hospital (CHUV) will be invited to participate. In addition, women with GDM who are followed up by private diabetologists and gynaecologists in the Vaud canton will be invited to participate following a similar procedure. Using the new criteria of the IADPSG, the expected prevalence of GDM in this population is at least ~10% [3]. Women with T1/T2D will not be included.

For peer review only - http://bmjopen.bmj.com/site/about/guidelines.xhtml

Study population

Inclusion criteria: Pregnant women aged 18 years or older, with or without GDM at 24-32 weeks of gestation, and understanding French or English; exclusion criteria: Women on strict bed-rest, with pre-existing DM or severe mental disorders precluding participation.

Study outcomes

Primary outcomes are surrogate markers of CVD and atherosclerosis at birth, that is, LVMI and cIMT. Secondary outcomes are foetal cardiovascular structure and function (notably LVMI and liver volume) and potential neonatal cardiovascular risk factors (including cord blood lipid levels, glycaemia, insulin, hs-CRP, etc.).

Data collection and visits

During pregnancy, in the pre-recruitment phase, all pregnant women without pre-existing DM, attending the CHUV antenatal care clinic, have a fasting blood glucose measurement between the 24th and 28th weeks of gestation. GDM is diagnosed if fasting blood glucose is equal to or above 5.1 mmol/l. If fasting blood glucose is equal to or above 4.4 and below 5.1 mmol/l, pregnant women have a 2-hour 75-g OGTT and GDM will be diagnosed based on the new criteria of the IADPSG [3, 9]. Women screened by private obstetricians in the Vaud canton undergo either the same procedure or directly a 2-hour 75-g OGTT. Considering the variability in patients' compliance and time of enrolment for prenatal care, all eligible women, having been screened for GDM before 32 weeks of gestation, will be invited to participate (see inclusion and exclusion criteria above).

The participation in the study is voluntary and involves a sequence of events and measurements as summarized in Table 1.

At the first study visit (V1), scheduled between the 24th and 32nd weeks of gestation, a baseline assessment of the mothers will be conducted through means of self-report questionnaires and a researcher-administrated interview, weight, height and blood pressure measurements, and a maternal blood sampling. This will provide information notably on the socioeconomic status, general health status, family medical history of CVD or diabetes, smoking and drinking habits, and several cardio-metabolic biomarkers, all of which will be treated as potential confounding factors. If available, paternal data on the family history of CVD or diabetes will be also collected at the first study visit.

At the second visit (V2), between the 30th and 34th weeks of gestation, a foetal echography will be performed to assess the foetal cardiac structure (including LVMI) and function, the foeto-maternal circulation, and the hepatic volume.

In women with GDM, information about treatment and blood glucose measurements will be recorded during the whole pregnancy.

At birth (V3), maternal characteristics (including mode of delivery, gravidity, parity, etc.) and neonatal characteristics (including gestational age, sex, weight, length, etc.) will be assessed. A sample of umbilical cord blood will be collected to measure various biomarkers, including glucose, insulin, total cholesterol, LDL-cholesterol, HDL-cholesterol, triglycerides, uric acid, creatinine, hs-CRP, and various miRNA.

For peer review only - http://bmjopen.bmj.com/site/about/guidelines.xhtml

An echocardiography will be done to assess cardiac structure (including LVMI) and function 2-7 days after birth (V4). Carotid intima-media thickness (IMT) will be measured to assess vascular structure.

Table 1. Schedule of events and main assessments. WG: weeks of gestation. CVD: cardiovascular diseases, N/A: not applicable.

	Visit	Period (WG)	Mother	Foetus/Newborn
Pregnancy	V1 (Baseline)	24th-32nd	• Information notably on socio-economic characteristics, general health, family history of CVD or diabetes, and smoking and drinking habits<br>• Weight and height measurements<br>• 3 blood pressure measurements<br>• Blood sampling	• N/A
	V2	30th-34th	• Weight<br>• 3 blood pressure measurements	• Foetal cardiac echography
Delivery or Post-delivery	V3	Birth	• Blood sampling	• Cord blood sampling, newborn weight, length and other birth-related information
	V4	2-7 days after birth	• Weight<br>• 3 blood pressure measurements	• Neonatal cardiac echography and carotid echography

Measurements methods

Maternal clinical assessment

Maternal body weight and blood pressure (3 readings at 2-minute intervals with a clinically validated [35] oscillometric sphygmomanometer (OMRON HEM-907) [36, 37]) will be measured during pregnancy (V1, V2) and in postpartum (V4). Clinical chemistry (including glucose, HbA1c, insulin, total cholesterol, LDL-cholesterol, HDL-cholesterol, triglycerides, uric acid, and creatinine) will be assessed at the first study visit (V1) and blood will be collected for further potential analyses. All samples will be managed and stored in a biobank. The clinical data assessed as part of the usual care of the mother and potentially influencing the outcomes of interest will be collected. Also, information on socio-economic characteristics, general health, family history of CVD or diabetes, smoking and drinking habits, etc., will be gathered using a self-report questionnaire and a researcher-administrated interview.

Offspring clinical assessment

The height and weight of the neonate will be measured and the growth reference charts of the Swiss Society of Paediatrics will be used [38, 39]. Gestational age will be determined using either the date of the last menstrual period or the first trimester ultrasound dating. Clinical chemistry (including glucose, total cholesterol, LDL-cholesterol, HDL-cholesterol, triglycerides, insulin, uric acid, hs-CRP, mi-RNA) will be assessed at birth using cord blood. We will explore the profile of circulating non-coding RNAs in offspring of women with GDM and without GDM to assess the involvement of epigenetic mechanisms in cardiovascular alterations [40]. Blood for

For peer review only

For peer review only - http://bmjopen.bmj.com/site/about/guidelines.xhtml

future analyses will be stored in a biobank. The clinical data assessed as part of the usual care of the offspring and potentially influencing the outcomes of interest will be collected.

Foetal echography

This exam will be performed between the 30th and 34th weeks of gestation by two experienced ultrasonographers (YM, YV), blinded to the maternal glycaemic status, using a Voluson E10 (General Electric Medical Systems, ZIPF, Australia) equipped with a 2–8 MHz convex array sector transducer [41]. A systematic echography will be conducted to detect structural and functional anomalies following international recommendations [42, 43]. Anthropometric measurements and foetal well-being parameters will be measured [44]. Observed foetal weight (FW) will be calculated using the Hadlock formula based on the head circumference, biparietal diameter, abdominal circumference, and femur length [45]. The gestational age-adjusted expected FW will be calculated [46] and the discordance with observed FW will be determined as (observed FW-expected FW)/expected FW. This discordance will be used to quantify appropriateness of foetal growth. Then, a detailed echocardiography of the foetal liver, cardiovascular system and of the materno-foetal circulation will be conducted including bidimensional (2D), M-Mode (M), Doppler (D) and tridimensional (3D) imaging of the following structures: cardiac 4 chambers (2D), RV and LV short axis (2D, M), tricuspid, pulmonary, mitral and aortic valve (2D, D), umbilical vein, ductus venosus, patent ductus arteriosus, aortic isthmus, umbilical arteries and maternal uterine arteries (2D, D) and liver (2D, 3D) [47-50]. This will allow the assessment of the LV posterior wall and septal thickness, the LV mass [51], the LV and RV systolic and diastolic function, the cardiothoracic ratio, the placental and foetal vascular resistances, the cardiac output and liver volume. Data will be digitally stored to allow off-line analyses. Measures will be expressed as z-scores based on gestational age to allow comparison between foetuses.

Neonatal cardiac echography

This exam will be performed 2-7 days after birth (before the mother and the newborn leave the clinic) by two experienced ultrasonographers (NS, SDB) blinded to the maternal glycaemic status and to the foetal echocardiogram. Echocardiography will be performed on a Philips EPIC echocardiogram with a S8-3 or S12 MHz transducer, digitally recorded (Xcelera, Philips) and analyzed off-line. Standard echocardiography including 2D, M-mode, colour and spectral Doppler according to the ASE will be performed [52, 53]. Measurements will be performed according to the standard of the ASE [52, 53] including: 1) right and left atrial chamber sizes; 2) LV size, volume and function; 3) LV mass; 4) LV and RV systolic and diastolic function. Z scores based on BSA calculated by the Haycock formula will be used to express these measurements [54-56].

Neonatal carotid echography

This exam will be performed 2-7 days after birth (before the mother and the newborn leave the clinic) by two experimented ultrasonographers (NS, SDB) blinded to the maternal glycaemic status. Carotid IMT measurement will be performed on a Philips EPIC echocardiograph (Philips Medical, Netherland) with a L 11-5 MHz high-resolution linear array transducer, recorded on a digital system (QLab, Philips Medical Netherlands). Image acquisition will be done according to the standard of the American Heart Association (AHA) Atherosclerosis, Hypertension and Obesity in Youth Committee of the Council on Cardiovascular Disease in the Young [31]. Image analyses will include: 1) far-wall cIMT; 2) calculation of the mean of maximal cIMT measurements; 3) calculation of carotid stiffness. Measurements of cIMT will be expressed in

For peer review only - http://bmjopen.bmj.com/site/about/guidelines.xhtml

mm as mean +/- SD and compared with normal cIMTs [57, 58]. We have recently conducted a study to show that the measurement of cIMT was feasible in young non-sedated infants. This study conducted among 81 infants less than 1 year of age confirmed that cIMT was measurable with a high inter-observer reliability (coefficient of variation: 5.9%)[59].

Anticipated recruitment

Recruitment has begun in September 2016. About 3200 deliveries per year occur at our hospital and about 250 pregnant women are followed for GDM at our specialised clinic. A relatively large share of patients consists of immigrants who do not speak English or French and will not be eligible for participation. We therefore expect to include up to 2 pregnant women per week, hence to include the 200 subjects in 30 months. A research coordinator (SE) and a PhD student (AME) will be responsible to screen all women for eligibility and to ensure follow-up throughout the study duration. Recruitment will be facilitated by the high support of obstetricians at the CHUV. We will also benefit of the new specialized GDM clinic of the CHUV where every pregnant woman who screened positive for GDM at the CHUV antenatal care clinic is followed up. *MySweetHeart Cohort* is linked to *MySweetHeart Trial* (clinicaltrials.gov: NCT02890693; led by Prof Jardena Puder and PD Dr Antje Horsch), a randomized controlled trial led by this new specialized GDM clinic designed to assess the effect of a multidimensional interdisciplinary lifestyle and psychosocial intervention to improve the cardio-metabolic and mental health of women with GDM and their offspring. Little loss to follow-up is expected because measurements are made during pregnancy and shortly after delivery. Nevertheless, to minimise loss to follow-up, most appointments are made on the days of regular follow-up visits and the postnatal echocardiography is scheduled before the mother and the baby leave the hospital.

Sample size

Assuming that LVMI will be 30 g/m$^{2.7}$ [SD: 4.5] in children of mothers without GDM [60] compared to 32 g/m$^{2.7}$ [SD: 4.5] in children of women with GDM, the required sample size is 80 women with GDM and 80 women without GDM to have a statistical significant difference with a power of 80% and an alpha-level set at 0.05 (two-sided). This sample size is also sufficient to observe statistical significant differences in cIMT at birth if we assume that cIMT at birth is 0.44 mm [SD: 0.04] [31] in newborn of women with GDM and 0.42 mm [SD: 0.04] in newborn of women without GDM. In the worst case scenario, we could lose up to 20% of participants between inclusion and delivery. Therefore, we plan to include 100 pregnant women with GDM and 100 women without GDM.

Statistical analysis

The association between exposure (GDM/no GDM) and the (continuous) outcomes will be estimated with linear regression analyses. Adjustment for potential confounding factors (maternal characteristics such as age, BMI, blood pressure, socio-economic status, personal history of CVD; treatment for GDM during pregnancy) will be done. To assess the direct and indirect (through mediators) effects of GDM on the outcomes, causal mediation analyses will be conducted [61, 62]. Because LGA is a potential mediator of the effect of GDM on offspring outcomes [63], analyses adjusted on birth weight will be conducted to estimate the indirect effect of GDM on the outcomes **(Figure 1)**. We will also consider the effect of treatment which will be treated as a mediator between GDM, LGA, and outcomes.

---FIGURE 1---

For peer review only - http://bmjopen.bmj.com/site/about/guidelines.xhtml

ETHICS AND DISSEMINATION

The study poses little to no risk to participants and their offspring. Signed informed consent is obtained from all participating women. Participation in the study does not interfere with the typical care patients receive during pregnancy and after delivery. Clinicians in the obstetrical clinic provide clinical follow-up and, if necessary, participants are referred on for additional clinical management.

Results from this study will be disseminated at regional and international conferences and in peer-reviewed journals.

SIGNIFICANCE AND OUTLOOK

The current high prevalence of maternal obesity and associated hyperglycaemic disorders may lead to a perpetuating intergenerational cycle of increasing obesity, metabolic and cardiovascular disorders. Well-designed prospective studies are necessary to better quantify the actual impact of maternal hyperglycaemic disorders on offspring's health and to identify the potential underlying mechanisms. Despite recent significant advances in Developmental Origin of Health and Disease and foetal programming research, major challenges have to be addressed, notably by better objectively characterizing both early life exposure and cardio-metabolic outcomes [23].

Indeed, many past studies on the foetal programming of cardio-metabolic disorders suffered from some methodological flaws including confounding, no or poor standardization of exposure and outcome assessment, and selection bias. Our prospective population-based study in pregnant women whose offspring is examined during foetal life and at birth avoids some of these issues. The long period needed for CVD to develop could be a limitation, but this will be overcome in our study by measuring surrogate outcomes (including LVMI and cIMT) that are well-established precursors of CVD [31]. In addition, a long-term follow-up of the offspring is planned (not funded yet) for further assessment of their cardiovascular health.

By looking at the foeto-maternal circulation, foetal cardiovascular system and liver, and foetal growth and CVD risk markers at birth, we will acquire unique data to gain insight in the early mechanisms underlying the development of CVD and metabolic diseases, and on the role of maternal hyperglycaemic disorders. GDM is an opportunity for timely intervention for the prevention of cardio-metabolic disorders in mothers and their offspring [9]. With the new criteria to define GDM, a growing proportion of women will be concerned by this disorder. Having a better understanding of the clinical impact of maternal hyperglycaemic disorders on offspring's health early in life is urgently needed to inform strategies for the early prevention of CVD.

Acknowledgements

Not applicable.

Collaborators

The following are members of *MySweetHeart Research Group* (including the authors of the present article), listed in alphabetical order: Pascal Bovet, MD MPH, Prof. (Institute of Social and Preventive Medicine (IUMSP), Lausanne University Hospital, Lausanne, Switzerland; pascal.bovet@chuv.ch), Arnaud Chiolero, MD PhD, Senior lecturer (Institute of Social and Preventive Medicine (IUMSP), Lausanne University Hospital, Lausanne, Switzerland;

For peer review only - http://bmjopen.bmj.com/site/about/guidelines.xhtml

Department of Epidemiology, Biostatistics, and Occupational Health, McGill University, Montreal Canada; Institute of Primary Health Care (BIHAM), University of Bern, Switzerland; arnaud.chiolero@chuv.ch), Stefano Di Bernardo, MD, Lecturer (Pediatric Cardiology Unit, Woman-Mother-Child Department, Lausanne University Hospital, Lausanne, Switzerland; stefano.di-bernardo@chuv.ch), Adina Mihaela Epure, MD (Pediatric Cardiology Unit, Woman-Mother-Child Department; Institute of Social and Preventive Medicine (IUMSP), Lausanne University Hospital, Lausanne, Switzerland; adina-mihaela.epure@chuv.ch), Sandrine Estoppey, M.Sc. (Institute of Social and Preventive Medicine (IUMSP), Lausanne University Hospital, Lausanne, Switzerland; sandrine.estoppey@chuv.ch), Leah Gilbert, M.Sc. (Division of Endocrinology, Diabetes and Metabolism, Department of Medicine, Lausanne University Hospital, Lausanne, Switzerland; leah.gilbert@chuv.ch), Justine Gross, HES (Division of Endocrinology, Diabetes and Metabolism, Department of Medicine, Lausanne University Hospital, Lausanne, Switzerland; justine.gross@chuv.ch), Antje Horsch, DClinPsych, Senior Lecturer, Woman-Mother-Child Department, Lausanne University Hospital, Lausanne, Switzerland; antje.horsch@chuv.ch), Bengt Kayser, PhD, Prof. (Institute of Sport Sciences, University of Lausanne; bengt.kayser@unil.ch), Stefano Lanzi, PhD (Division of Endocrinology, Diabetes and Metabolism, Department of Medicine, Lausanne University Hospital, Lausanne, Switzerland; stefano.lanzi@chuv.ch), Yvan Mivelaz, MD, Lecturer (Pediatric Cardiology Unit, Woman-Mother-Child Department, Lausanne University Hospital, Lausanne, Switzerland; yvan.mivelaz@chuv.ch), Jardena J. Puder, MD, Prof. (Division of Endocrinology, Diabetes and Metabolism, Department of Medicine, Lausanne University Hospital, Lausanne, Switzerland; jardena.puder@chuv.ch), Nicole Sekarski, MD, Prof. (Pediatric Cardiology Unit, Woman-Mother-Child Department, Lausanne University Hospital, Lausanne, Switzerland; nicole.sekarski@chuv.ch), Umberto Simeoni, MD, Prof. (DOHad Laboratory, Pediatrics Division, Woman-Mother-Child Department, Lausanne University Hospital, Lausanne, Switzerland; umberto.simeoni@chuv.ch), Yvan Vial, MD, Senior lecturer (Obstetrics and Gynecology Division, Woman-Mother-Child Department, Lausanne University Hospital, Lausanne, Switzerland; yvan.vial@chuv.ch).

Contributors

NS, AC, SDB and YM designed the study with input from all other authors. SDB and YM drafted the manuscript and contributed equally to the present work. US, PB, AME, SE, YV, and AC significantly contributed to the establishment and refinement of study procedures and critically revised the manuscript. All authors approved the final version of the manuscript.

Funding

This study is funded by the Swiss National Science Foundation (Project 32003B-163240). The study protocol has been peer-reviewed by national and international experts before being funded.

Competing interests

None declared.

Ethics approval

The study has been approved by the state Human Research Ethics Committee of the Canton de Vaud (study number 2016-00745).

For peer review only - http://bmjopen.bmj.com/site/about/guidelines.xhtml

Provenance and peer review

Not commissioned; externally peer reviewed.

Data sharing statement

It is the intention of the authors that once *MySweetHeart Cohort* is completed and the research articles emerging from the study have been published, the data set supporting the results and conclusions presented in the respective research articles to be made available in the DATA@IUMSP repository, https://data.iumsp.ch.

Open access

This is an Open Access article distributed in accordance with the terms of the Creative Commons Attribution (CC BY 4.0) license, which permits others to distribute, remix, adapt and build upon this work, for commercial use, provided the original work is properly cited. See: http://creativecommons.org/licenses/by/4.0/

For peer review only

For peer review only - http://bmjopen.bmj.com/site/about/guidelines.xhtml

FIGURE LEGEND

Figure 1. Hypothetical and highly-simplified causal relationships between gestational diabetes mellitus (GDM) and cardio-metabolic disorders. Two pathways can be hypothesized: 1) a direct effect of GDM on offspring's cardio-metabolic disorders; 2) an indirect effect through the mediator large for gestational age (LGA). It means that if LGA was prevented, part of the effect of GDM on cardio-metabolic disorders would be prevented. Several confounding factors (such as maternal obesity, weight gain during pregnancy, smoking, socio-economic status, or family history of cardio-metabolic diseases) have to be accounted.

For peer review only

For peer review only - http://bmjopen.bmj.com/site/about/guidelines.xhtml

REFERENCES

1 Hunt KJ, Schuller KL. The increasing prevalence of diabetes in pregnancy. *Obstet Gynecol Clin North Am* 2007;34(2):173-99.
2 Albrecht SS, Kuklina EV, Bansil P, et al. Diabetes trends among delivery hospitalizations in the U.S., 1994-2004. *Diabetes Care* 2010;33(4):768-73.
3 Metzger BE, Gabbe SG, Persson B, et al. International association of diabetes and pregnancy study groups recommendations on the diagnosis and classification of hyperglycemia in pregnancy. *Diabetes Care* 2010;33(3):676-82.
4 Reece EA, Leguizamon G, Wiznitzer A. Gestational diabetes: the need for a common ground. *Lancet* 2009;373(9677):1789-97.
5 Reece EA. The fetal and maternal consequences of gestational diabetes mellitus. *J Matern Fetal Neonatal* 2010;23(3):199-203.
6 Noussitou P, Monbaron D, Vial Y, et al. Gestational diabetes mellitus and the risk of metabolic syndrome: a population-based study in Lausanne, Switzerland. *Diabetes Metab* 2005;31(4 Pt 1):361-9.
7 Landon MB, Mele L, Spong CY, et al. The relationship between maternal glycemia and perinatal outcome. *Obstet Gynecol* 2011;117(2 Pt 1):218-24.
8 Ullmo S, Vial Y, Di Bernardo S, et al. Pathologic ventricular hypertrophy in the offspring of diabetic mothers: a retrospective study. *Eur Heart J* 2007;28(11):1319-25.
9 Nolan CJ. Controversies in gestational diabetes. *Best Pract Res Clin Obstet Gynaecol* 2011;25(1):37-49.
10 Metzger BE, Lowe LP, Dyer AR, et al. Hyperglycemia and adverse pregnancy outcomes. *N Engl J Med* 2008;358(19):1991-2002.
11 Nolan CJ, Damm P, Prentki M. Type 2 diabetes across generations: from pathophysiology to prevention and management. *Lancet* 2011;378(9786):169-81.
12 Crowther CA, Hiller JE, Moss JR, et al. Effect of treatment of gestational diabetes mellitus on pregnancy outcomes. *N Engl J Med* 2005;352(24):2477-86.
13 Landon MB, Spong CY, Thom E, et al. A multicenter, randomized trial of treatment for mild gestational diabetes. *N Engl J Med* 2009;361(14):1339-48.
14 Leary J, Pettitt DJ, Jovanovic L. Gestational diabetes guidelines in a HAPO world. *Best Pract Res Clin Endocrinol Metab* 2010;24(4):673-85.
15 Landon MB. Is there a benefit to the treatment of mild gestational diabetes mellitus? *Am J Obstet Gynecol* 2010;202(6):649-53.
16 Hillier TA, Vesco KK, Pedula KL, et al. Screening for gestational diabetes mellitus: a systematic review for the U.S. Preventive Services Task Force. *Ann Intern Med* 2008;148(10):766-75.
17 Tieu J, Middleton P, McPhee AJ, et al. Screening and subsequent management for gestational diabetes for improving maternal and infant health. *Cochrane Database Syst Rev* 2010(7):CD007222.
18 Veille JC, Hanson R, Sivakoff M, et al. Fetal cardiac size in normal, intrauterine growth retarded, and diabetic pregnancies. *Am J Perinatol* 1993;10(4):275-9.
19 Yajnik CS. Fetal programming of diabetes: still so much to learn! *Diabetes Care* 2010;33(5):1146-8.
20 Hanson MA, Gluckman PD. Early developmental conditioning of later health and disease: physiology or pathophysiology? *Physiol Rev* 2014;94(4):1027-76.
21 Simeoni U, Yzydorczyk C, Siddeek B, et al. Epigenetics and neonatal nutrition. *Early Hum Dev* 2014;90 Suppl 2:S23-4.
22 Gluckman PD, Hanson MA, Cooper C, et al. Effect of in utero and early-life conditions on adult health and disease. *N Engl J Med* 2008;359(1):61-73.

For peer review only - http://bmjopen.bmj.com/site/about/guidelines.xhtml

23 Hivert MF, Perng W, Watkins SM, et al. Metabolomics in the developmental origins of obesity and its cardiometabolic consequences. *J Dev Orig Health Dis* 2015:1-14.
24 Kiserud T. Diabetes in pregnancy: scanning the wrong horizon? *Ultrasound Obstet Gynecol* 2010;36(3):266-7.
25 Kessler J, Rasmussen S, Godfrey K, et al. Venous liver blood flow and regulation of human fetal growth: evidence from macrosomic fetuses. *Am J Obstet Gynecol* 2011;204(5):429.
26 Ebbing C, Rasmussen S, Kiserud T. Fetal hemodynamic development of macrosomic growth. *Ultrasound Obstet Gynecol* 2011;38(3):303-8.
27 Tchirikov M, Kertschanska S, Sturenberg HJ, et al. Liver blood perfusion as a possible instrument for fetal growth regulation. *Placenta* 2002;23 Suppl A:S153-8.
28 Mirghani H, Zayed R, Thomas L, et al. Gestational diabetes mellitus: fetal liver length measurements between 21and 24 weeks' gestation. *J Clin Ultrasound* 2007;35(1):34-7.
29 Anderson NG, Notley E, Graham P, et al. Reproducibility of sonographic assessment of fetal liver length in diabetic pregnancies. *Ultrasound Obstet Gynecol* 2008;31(5):529-34.
30 Sorof JM, Alexandrov AV, Cardwell G, et al. Carotid artery intimal-medial thickness and left ventricular hypertrophy in children with elevated blood pressure. *Pediatrics* 2003;111(1):61-6.
31 Urbina EM, Williams RV, Alpert BS, et al. Noninvasive assessment of subclinical atherosclerosis in children and adolescents: recommendations for standard assessment for clinical research: a scientific statement from the American Heart Association. *Hypertension* 2009;54(5):919-50.
32 McGill HC, Jr., McMahan CA, Gidding SS. Preventing heart disease in the 21st century: implications of the Pathobiological Determinants of Atherosclerosis in Youth (PDAY) study. *Circulation* 2008;117(9):1216-27.
33 McGill HC, McMahan CA, Gidding SS. Are pediatricians responsible for prevention of adult cardiovascular disease? *Nat Clin Pract Cardiovasc Med* 2009;6(1):10-1.
34 Simonetti GD, RO VONV, Wuhl E, et al. Ambulatory arterial stiffness index is increased in hypertensive childhood disease. *Pediatr Res* 2008;64(3):303-7.
35 Chiolero A, Paradis G, Lambert M. Accuracy of oscillometric devices in children and adults. *Blood Press* 2010;19(4):254-9.
36 El Assaad MA, Topouchian JA, Darne BM, et al. Validation of the Omron HEM-907 device for blood pressure measurement. *Blood Press Monit* 2002;7(4):237-41.
37 White WB, Anwar YA. Evaluation of the overall efficacy of the Omron office digital blood pressure HEM-907 monitor in adults. *Blood Press Monit* 2001;6(2):107-10.
38 Braegger C, Jenni O, Konrad D, et al. Nouvelles courbes de croissance pour la Suisse. *Paediatrica* 2011;22:9-11.
39 de Onis M, Garza C, Victora CG, et al. The WHO Multicentre Growth Reference Study. *Food Nutr Bull* 2004;25(1 Suppl):3-84.
40 Fichtlscherer S, De Rosa S, Fox H, et al. Circulating microRNAs in patients with coronary artery disease. *Circ Res* 2010;107(5):677-84.
41 Salomon LJ, Alfirevic Z, Berghella V, et al. Practice guidelines for performance of the routine mid-trimester fetal ultrasound scan. *Ultrasound Obstet Gynecol* 2011;37(1):116-26.
42 Lee W, Allan L, Carvalho JS, et al. ISUOG consensus statement: what constitutes a fetal echocardiogram? *Ultrasound Obstet Gynecol* 2008;32(2):239-42.
43 Tutschek B, Schmidt KG. Techniques for assessing cardiac output and fetal cardiac function. *Semin Fetal Neonatal Med* 2011;16(1):13-21.
44 Papageorghiou AT, Ohuma EO, Altman DG, et al. International standards for fetal growth based on serial ultrasound measurements: the Fetal Growth Longitudinal Study of the INTERGROWTH-21st Project. *Lancet* 2014;384(9946):869-79.
45 Hadlock FP, Harrist RB, Sharman RS, et al. Estimation of fetal weight with the use of head, body, and femur measurements--a prospective study. *Am J Obstet Gynecol* 1985;151(3):333-7.

For peer review only

For peer review only - http://bmjopen.bmj.com/site/about/guidelines.xhtml

46 Salomon LJ, Bernard JP, Ville Y. Estimation of fetal weight: reference range at 20-36 weeks' gestation and comparison with actual birth-weight reference range. *Ultrasound Obstet Gynecol* 2007;29(5):550-5.
47 Gagnon C, Bigras JL, Fouron JC, et al. Reference Values and Z Scores for Pulsed-Wave Doppler and M-Mode Measurements in Fetal Echocardiography. *J Am Soc Echocardiogr* 2016;29(5):448-60.
48 Boito SM, Struijk PC, Ursem NT, et al. Assessment of fetal liver volume and umbilical venous volume flow in pregnancies complicated by insulin-dependent diabetes mellitus. *BJOG* 2003;110(11):1007-13.
49 Ebbing C, Rasmussen S, Kiserud T. Middle cerebral artery blood flow velocities and pulsatility index and the cerebroplacental pulsatility ratio: longitudinal reference ranges and terms for serial measurements. *Ultrasound Obstet Gynecol* 2007;30(3):287-96.
50 Gomez O, Figueras F, Fernandez S, et al. Reference ranges for uterine artery mean pulsatility index at 11-41 weeks of gestation. *Ultrasound Obstet Gynecol* 2008;32(2):128-32.
51 Reichek N, Helak J, Plappert T, et al. Anatomic validation of left ventricular mass estimates from clinical two-dimensional echocardiography: initial results. *Circulation* 1983;67(2):348-52.
52 Lai WW, Geva T, Shirali GS, et al. Guidelines and standards for performance of a pediatric echocardiogram: a report from the Task Force of the Pediatric Council of the American Society of Echocardiography. *J Am Soc Echocardiogr* 2006;19(12):1413-30.
53 Lopez L, Colan SD, Frommelt PC, et al. Recommendations for quantification methods during the performance of a pediatric echocardiogram: a report from the Pediatric Measurements Writing Group of the American Society of Echocardiography Pediatric and Congenital Heart Disease Council. *J Am Soc Echocardiogr* 2010;23(5):465-95.
54 Daubeney PE, Blackstone EH, Weintraub RG, et al. Relationship of the dimension of cardiac structures to body size: an echocardiographic study in normal infants and children. *Cardiol Young* 1999;9(4):402-10.
55 Pettersen MD, Du W, Skeens ME, et al. Regression equations for calculation of z scores of cardiac structures in a large cohort of healthy infants, children, and adolescents: an echocardiographic study. *J Am Soc Echocardiogr* 2008;21(8):922-34.
56 Haycock GB, Schwartz GJ, Wisotsky DH. Geometric method for measuring body surface area: a height-weight formula validated in infants, children, and adults. *J Pediatr* 1978;93(1):62-6.
57 Sass C, Herbeth B, Chapet O, et al. Intima-media thickness and diameter of carotid and femoral arteries in children, adolescents and adults from the Stanislas cohort: effect of age, sex, anthropometry and blood pressure. *J Hypertens* 1998;16(11):1593-602.
58 Jourdan C, Wuhl E, Litwin M, et al. Normative values for intima-media thickness and distensibility of large arteries in healthy adolescents. *J Hypertens* 2005;23(9):1707-15.
59 Mivelaz Y, Di Bernardo S, Boulos Ksontini T, et al. Feasibility and reliability of carotid intima-media thickness measurements in nonsedated infants. *J Hypertens* 2016;34(11):2227-32.
60 Maggio AB, Aggoun Y, Marchand LM, et al. Associations among obesity, blood pressure, and left ventricular mass. *J Pediatr* 2008;152(4):489-93.
61 VanderWeele TJ, Mumford SL, Schisterman EF. Conditioning on intermediates in perinatal epidemiology. *Epidemiology* 2012;23(1):1-9.
62 Chiolero A, Kaufman JS, Paradis G. Why adjustment for current weight can bias the estimate of the effect of birth weight on blood pressure: shedding light using causal graphs. *J Hypertens* 2012;30(5):1042-5.
63 Vohr BR, Boney CM. Gestational diabetes: the forerunner for the development of maternal and childhood obesity and metabolic syndrome? *J Matern Fetal Neonatal* 2008;21(3):149-57.

For peer review only - http://bmjopen.bmj.com/site/about/guidelines.xhtml

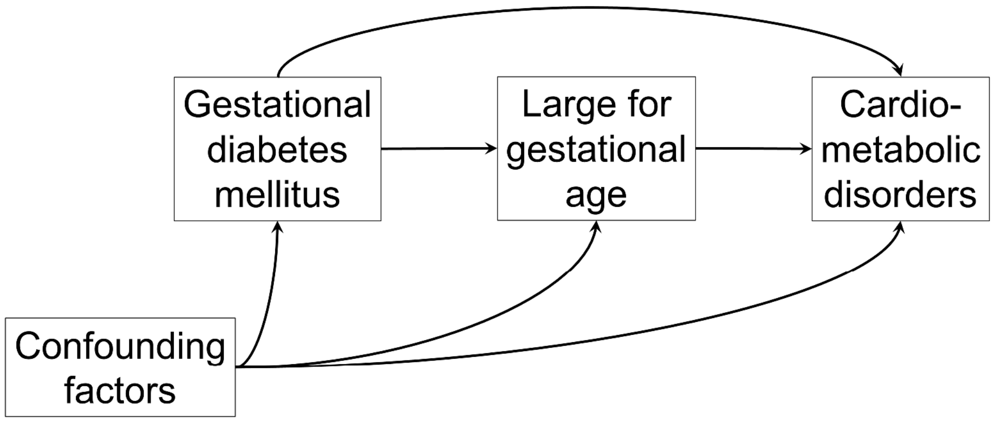

Hypothetical and highly-simplified causal relationships between gestational diabetes mellitus (GDM) and cardio-metabolic disorders. Two pathways can be hypothesized: 1) a direct effect of GDM on offspring's cardio-metabolic disorders; 2) an indirect effect through the mediator large for gestational age (LGA). It means that if LGA was prevented, part of the effect of GDM on cardio-metabolic disorders would be prevented. Several confounding factors (such as maternal obesity, weight gain during pregnancy, smoking, socio-economic status, or family history of cardio-metabolic diseases) have to be accounted.

338x190mm (300 x 300 DPI)

For peer review only - http://bmjopen.bmj.com/site/about/guidelines.xhtml
